# Effect of strain-induced anisotropy on magnetization dynamics in Y_3_Fe_5_O_12_ films recrystallized on a lattice-mismatched substrate

**DOI:** 10.1038/s41598-021-93308-3

**Published:** 2021-07-07

**Authors:** Adam Krysztofik, Sevgi Özoğlu, Robert D. McMichael, Emerson Coy

**Affiliations:** 1 Institute of Molecular Physics, Polish Academy of Sciences, ul. Smoluchowskiego 17, 60-179 Poznan, Poland; 2 Faculty of Physics, Adam Mickiewicz University, Uniwersytetu Poznanskiego 2, 61-614 Poznan, Poland; 3 Department of Physics, Graduate School of Natural and Applied Sciences, Hakkari University, 30000 Hakkari, Turkey; 4 National Institute of Standards and Technology, Gaithersburg, MD 20899 USA; 5 NanoBioMedical Centre, Adam Mickiewicz University, ul. Wszechnicy Piastowskiej 3, 61-614 Poznan, Poland

**Keywords:** Magnetic properties and materials, Ferromagnetism

## Abstract

We report on the correlation of structural and magnetic properties of Y_3_Fe_5_O_12_ (YIG) films deposited on Y_3_Al_5_O_12_ substrates using pulsed laser deposition. The recrystallization process leads to an unexpected formation of interfacial tensile strain and consequently strain-induced anisotropy contributing to the perpendicular magnetic anisotropy. The ferromagnetic resonance linewidth of YIG is significantly increased in comparison to a film on a lattice-matched Gd_3_Ga_5_O_12_ substrate. Notably, the linewidth dependency on frequency has a negative slope. The linewidth behavior is explained with the proposed anisotropy dispersion model.

## Introduction

In recent years, yttrium iron garnet (YIG) has been an intensively studied material for spintronic, photonic, and magnonic applications, mainly due to the low damping of magnetization precession^1–4^. Because of its potential applications in modern electronic devices, the research interest has been dominated by the development and processing of thin garnet films with perpendicular magnetic anisotropy^5^. Therefore, much scientific effort has been put into tuning the magnetic anisotropy of YIG films on the atomic level. Typical approaches include the tuning via yttrium sites substitution with other rare earth elements^6–11^, inducing interfacial strain due to lattice parameter mismatch^12–17^, ion implantation^18^, or reducing shape anisotropy by film patterning^19,20^.

The intended general effect of these approaches on anisotropic film properties is essentially realized and its impact on the magnetization dynamics is understood as well. For instance, in rare earth substituted or doped iron garnets one can expect an increase in Gilbert damping due to enhancement of spin–orbit coupling^7^. However, the mechanism of ferromagnetic resonance (FMR) linewidth broadening for YIG films is less understood. For the layers deposited on any substrate other than lattice-matched GGG, the FMR linewidth is significantly increased mainly due to strain, the polycrystalline structure of the films resulting in magnetocrystalline anisotropy field dispersion or extrinsic effects caused by surface crackings^21–25^. The joint effect of these contributions makes the linewidth analysis ambiguous and hinders further development of the material which is known for possessing the lowest magnetization losses^15,26^. Nonetheless, controlled growth of epitaxial and strained YIG films can provide an opportunity to investigate the linewidth contribution coming only from epitaxial strain.

In the FMR and spin pumping experiments of Wang et al.^12^, YIG films deposited on YAG exhibited exceptional crystalline quality, small surface roughness, and high spin mixing conductance of the interface with Pt. Nevertheless, the FMR linewidth of a bare, 73 nm thick YIG film on YAG was increased up to 8.4 mT when compared with YIG/GGG system. The authors suggested that the increase was likely related to the strain-induced defects in the films, although without further discussion. It was also reported that the films underwent a compressive strain which resulted in the easy-plane anisotropy contribution. The studies of YIG deposited on Sm_3_Ga_5_O_12_, (Gd_0.63_Y_2.37_)(Sc_2_Ga_3_)O_12_, Gd_3_(Sc_2_Ga_3_)O_12_ revealed, on the other hand, that tensile strain leads to the emergence of the perpendicular magnetic anisotropy (with easy out-of-plane axis), however, the magnetization damping of such strained films remained to be investigated and explained^13,14^.

Another important question concerns the effect of YIG thermal reconstruction on a lattice-mismatched substrate and its influence on the film properties. The deposition of amorphous material and subsequent recrystallization via annealing can provide technological advantages in terms of applicability of the lift-off technique for film patterning^19,27,28^. For YIG films deposited on GGG substrate using the pulsed laser deposition (PLD) technique, it was shown that the recrystallization does not significantly affect the structural or magnetic properties of the films, which can be the result of the rather low lattice mismatch of the YIG/GGG system^27^. It is however not clear if the same result can be accomplished for a strained YIG film on a lattice-mismatched substrate and if so, to which extent the functional properties of the films can be sustained.

Therefore, the aim of this study can be divided into two general objectives: to investigate the impact of the recrystallization process on structural properties of YIG deposited on YAG substrate and examine FMR linewidth broadening mechanisms for such a system. In this article, we will show that the recrystallization process of YIG films on YAG substrates is possible and that the lattice-mismatch of the substrates poses no impediment for its reconstruction. Moreover, we will show that the epitaxial strain follows unexpected tensile values, providing a unique opportunity for examining the perpendicular magnetic anisotropy. Finally, we provide a comprehensive interpretation for the linewidth broadening of the samples, based on the anisotropy dispersion model, with excellent agreement with our experimental results.

## Results and discussion

### Structural properties

**Figure 1:**
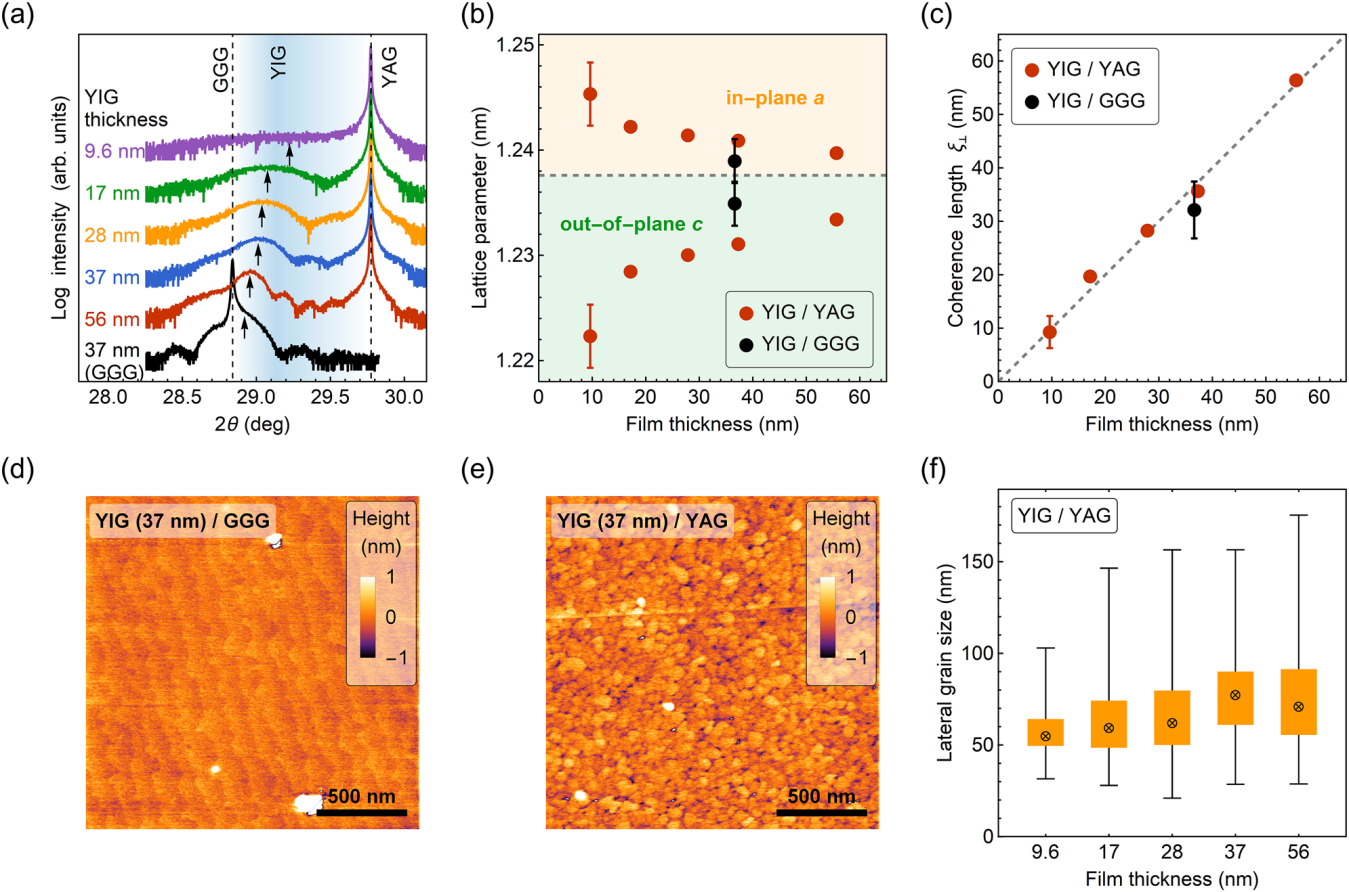
(**a**) X-ray ω-2θ diffraction patterns. (004) reflections of YIG are marked with arrows. The sharp peaks at 29.77° correspond to YAG (004) reflection, while the peak at 28.84° corresponds to GGG (004) reflection. (**b**) Thickness dependence of the determined out-of-plane lattice parameters $c$ and calculated in-plane lattice parameters $a$. The dashed line corresponds to the value of a bulk, cubic YIG. Error bars represent one standard deviation uncertainty. (**c**) Thickness dependence of the x-ray coherence length determined via Scherrer equation. The dashed line serves as a guide to the eye showing a one-to-one correlation of the values. (**d**) and (**e**) display surface morphologies of YIG films grown on YAG and GGG substrate. (**f**) Box-whisker plot of the grain size distributions. For each film thickness, the statistics are based on at least 300 grain measurements. The ⊗ marker corresponds to the median value, the bottom and the top box edges to 25th and 75th percentile, respectively, and the black lines to the upper and lower fence, illustrating extreme values in the tails of the distributions.

X-ray diffraction results point to an occurrence of a tensile strain in the films. As shown in Fig. 1a, a gradual shift in the position of YIG (004) reflection is observed when the thickness of the layer changes. For the thickest YIG film (56 nm) on YAG, the (004) reflection nearly coincides with one of the reference film deposited on the GGG substrate. As the film thickness decreases, the films experience a strain and the YIG out-of-plane (OP) lattice parameter $c$ approaches the lattice parameter of YAG substrate (1.2006 nm)^29^. Assuming that the volume of a unit cell is conserved, one can estimate in-plane (IP) lattice parameters according to $a = \sqrt {V_{{{\text{bulk}}}} /c}$, where $V_{{{\text{bulk}}}} =$ 1.2376^3^ nm^3^ is a volume of a bulk, cubic YIG unit cell. The comparison of the determined IP and OP lattice parameters shown in Fig. 1b, indicates the IP stretching of a unit cell.

The observed dependence of OP lattice parameter versus film thickness is reversed in comparison to YIG films deposited on YAG at high temperatures^12,30^. The recrystallization process of YIG from an amorphous phase on a lattice-mismatched substrate may therefore result in different epitaxial relations which are subsequently reflected in the magnetic properties. The correlation with magnetic properties is discussed in the Magnetic properties section. The dynamical aspect of the crystal lattice formation during post-deposition annealing remains, however, an open question still to be addressed since one would expect a compressive strain based on a comparison of the lattice parameters of YIG and YAG ($a_{{{\text{YAG}}}} < a_{{{\text{YIG}}}}$). Nonetheless, the study of Popova et al. showed, that the strain relaxation for lattice-mismatched garnets can be realized via coexistence of the so-called Vernier of misfit and the tilted growth mechanisms^31^. The first type of relaxation occurs when the bond strength within the film or the substrate is larger than the bonding between them. Such a mechanism alone can result in a relatively small residual strain^31^. The second type, however, leads to a tensile strain which is accommodated via rotation (tilting) of the film unit cells with respect to the substrate as well as via dislocations in close vicinity to the interface^31^.

Analysis of the Bragg reflection width for the investigated films indicates that the recrystallization occurred throughout the film thickness, although not many Laue oscillations are observed in the XRD spectra. The vertical size of a coherently scattering volume is evaluated by using Scherrer formula^32,33^:

1 $$\xi _{ \bot } = \lambda /\left( {{{\Gamma }}_{{2\theta }} \cos \theta } \right),$$

where $\xi _{ \bot }$ is the x-ray coherence length, $\lambda =$ 0.15406 nm is the Cu K_α_ radiation wavelength, ${{\Gamma }}_{{2\theta }}$ is the full width at half maximum intensity in the $2\theta$ direction. The determined values of $\xi _{ \bot }$ are in exact correspondence with film thicknesses and point to a high crystallinity of the films (Fig. 1c).

AFM surface topography maps show a distinct difference between YIG films grown on YAG and GGG substrate (Fig. 1d, e). While the film deposited on GGG exhibits terraces of the length ≈ 120 nm and height ≈ 0.4 nm, for the films deposited on YAG the recrystallization resulted in the formation of grains of different lateral sizes. The area $A$ of each distinguishable grain was measured and the grain diameter $d$ was estimated ($d = 2\sqrt {A/\pi }$). The statistics of grain size distribution are presented in Fig. 1f with the use of a box-whisker plot. No significant dependence on film thickness is observed and the median value of the grain size is ≈ 60 nm. It should be concurrently noted that the root-mean-square surface roughness (RMS) of the films is around 1 nm or less (Table 1). One can tentatively interpret the observed grains as corresponding to regions characterized by different growth mechanisms (Vernier of misfit and/or the tilted growth).

**Table 1:** Structural and magnetic properties of YIG films deposited on YAG and GGG (italic) substrate.

$t~$ (nm)	RMS (nm)	$c~$ (nm)	$a~$ (nm)	$\frac{{c - a}}{a}$ (%)	$M_{s} ~$ (kA/m)	$\mu _{0} H_{c} ~$ (mT)	$M_{{{\text{eff}}}} ~$ (kA/m)	$\mu _{0} H_{u} ~$ (mT)	$\mu _{0} {{\Gamma }}_{4} ~$ (mT)
9.6	0.4	1.2223	1.2453	− 1.85	–	–	–	–	–
17	1.0	1.2284	1.2422	− 1.11	127 ± 6	4.6 ± 0.3	68 ± 3	71.6 ± 3.8	0.27 ± 0.22
28	1.1	1.2300	1.2414	− 0.92	119 ± 8	5.1 ± 0.2	79 ± 1	57.8 ± 1.3	1.01 ± 0.08
37	0.3	1.2311	1.2409	− 0.79	123 ± 6	5.9 ± 0.3	95 ± 2	37.7 ± 2.5	1.33 ± 0.08
56	0.3	1.2334	1.2397	− 0.51	122 ± 6	5.7 ± 0.2	101 ± 1	30.2 ± 1.3	1.27 ± 0.04
*37*	*0.2*	*1.2349*	*1.2390*	− *0.33*	*131* ± *5*	*9.5* ± 0.*3*	*103* ± *1*	*27.6* ± *1.3*	*0.01* ± *0.03*

### Magnetic properties

**Figure 2:**
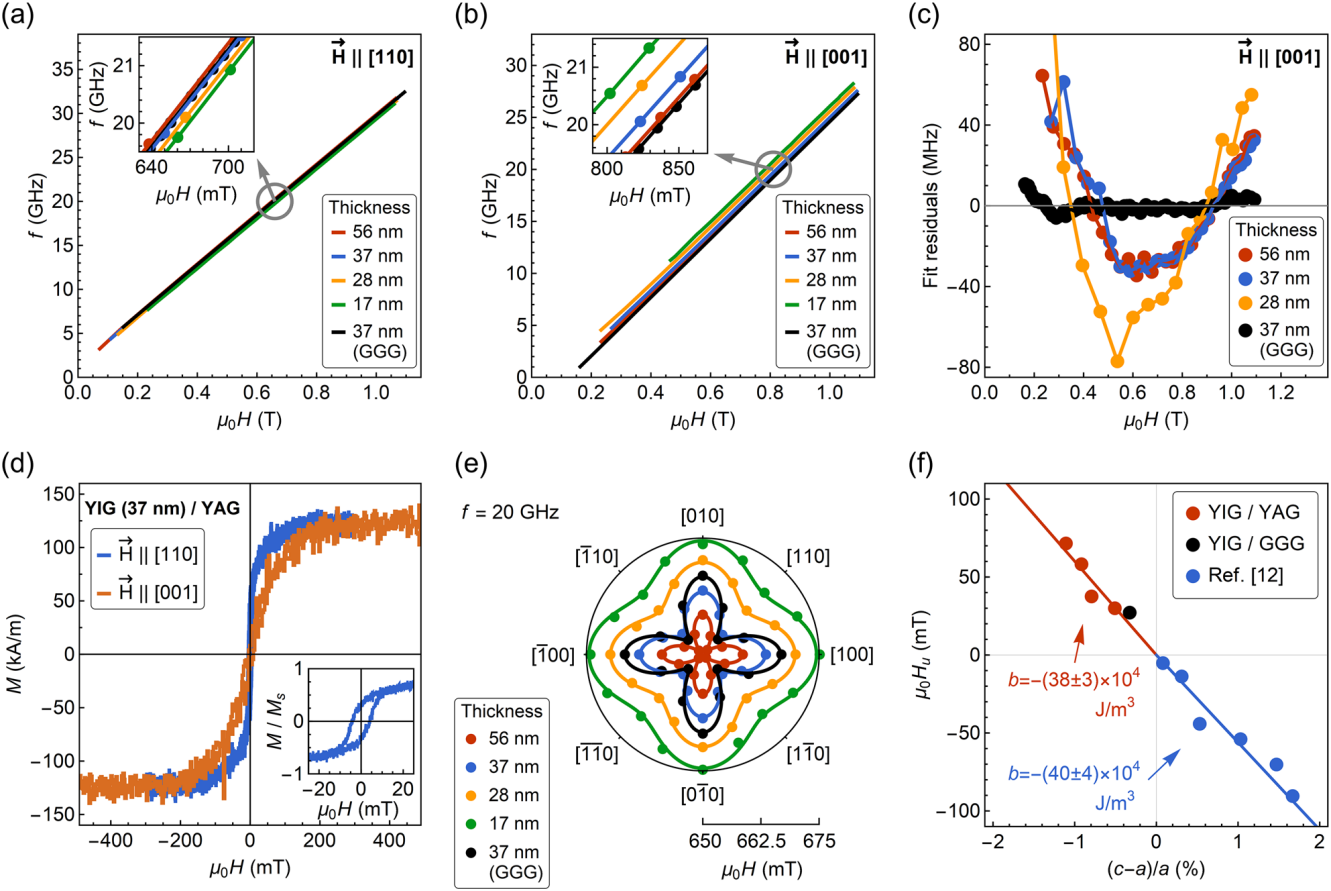
(**a**) and (**b**) show frequency versus resonance field dependencies for magnetic field applied along in-plane [110] and out-of-plane [001] directions, respectively. The insets display enlarged regions near $f =$ 20 GHz. (**c**) Fit residuals obtained from a fitting with Eq. (4) for the out-of-plane measurement configuration. Due to larger linewidth and lower signal-to-noise ratio, the residuals for the 17 nm thick film varied randomly in the range of ‒45 to 60 MHz, and therefore, have been omitted. (**d**) Hysteresis loops of 37 nm thick film deposited on YAG. Diamagnetic contribution from the substrate was subtracted from the raw data. Inset shows the enlarged view at the low fields for ${{\vec{H}}}$ || [110]. (**e**) In-plane angular dependence of the resonance field recorded at a constant frequency $f =$ 20 GHz. (**f**) Out-of-plane uniaxial anisotropy field as a function of the tetragonal distortion of YIG unit cell.

The saturation magnetization $M_{s}$ of the films has been found consistent across the set of samples (Table 1). The values at around 125 kA/m are congruent with previously reported 118 kA/m for the recrystallized YIG grown by us^27^, although slightly decreased in comparison to a bulk YIG at 140 kA/m. The small deviation from the bulk value can be understood as a result of subtle oxygen and cation nonstoichiometry^34^. Contrary to the YIG/GGG sample exhibiting coercivity of $\mu _{0} H =$ 0.4 mT and rectangular hysteresis loop measured along IP easy axis [110], the hysteresis loop for YIG film deposited on YAG, reveals increased coercivity of $\mu _{0} H =$ 4.5 mT and remanent to saturation magnetization ratio of $M_{r} /M_{s} =$ 0.4 (Fig. 2d). Additionally, a rounded saturation of the magnetization reversal curve for OP measurement is visible. The observed magnetostatic response has been found typical for the strained films^12–14,35,36^.

Angle-resolved FMR measurements indicate that the YIG unit cells are well-ordered in-plane. The results displayed in Fig. 2e clearly show that the films possess magnetocrystalline anisotropy with IP fourfold symmetry. By using the Kittel equation (see derivation in the Supplementary Information)^37^:

2 $$f = \frac{{\mu _{0} \gamma }}{{2\pi }}\sqrt {\left( {H + H_{c} ~\cos ~4\theta _{H} } \right)\left( {H + \frac{1}{4}H_{c} \left( {3 + ~\cos ~4\theta _{H} } \right) + M_{{{\text{eff}}}} } \right)} ,$$

3 $$M_{{{\text{eff}}}} = M_{s} - H_{u} ,$$

the magnetocrystalline anisotropy field $H_{c}$ was evaluated from the dependence of resonance magnetic field $H$ versus IP angle $\theta _{H}$ at a constant frequency $f =$ 20.000 GHz. In Eq. (2), $\gamma$ is the gyromagnetic ratio and $H_{u}$ is the uniaxial, out-of-plane anisotropy field. The obtained values of $\mu _{0} H_{c}$ (juxtaposed in Table 1), range from 4.6 to 9.5 mT, in agreement with the data reported for bulk and thin YIG films (1.2–8.7 mT)^12,27,34,38,39^.

To investigate the influence of strain on magnetic anisotropy, we use broadband FMR measurements. Frequency versus resonance magnetic field dependence is analyzed for the data taken along easy IP direction [110] (Fig. 2a). The values of $M_{{{\text{eff}}}}$ are determined from the fitting to Eq. (2) with $\theta _{H} =$ 45°. Subsequently, by using Eq. (3), uniaxial OP anisotropy field $H_{u}$ is calculated assuming constant $M_{s} =$ 125 kA/m following the results obtained with VSM magnetometry.

The strain-induced anisotropy field $H_{u}$ scales linearly with the tetragonal distortion of the YIG unit cell $\left( {c - a} \right)/a$, and the observed tensile strain results in positive values of the anisotropy field $H_{u}$ (Fig. 2f). In comparison to YIG films deposited on YAG at high temperatures, which experienced a compressive strain, the negative values of $H_{u}$ were reported, that corresponds to the easy-plane anisotropy contribution^12^. This shows that the strain tunability of the YIG anisotropy depends not only on the film thickness but also on the crystal growth conditions. From the slope of $\mu _{0} H_{u} = b\frac{2}{{M_{s} }} \cdot \frac{{c - a}}{a}$ dependence^9,12^, the magnetoelastic constant is evaluated $b =$ ‒(38 ± 5) × 10^4^ J/m^3^, which is within the range ‒(26 to 47) × 10^4^ J/m^3^ reported for bulk and thin-film YIG^12,16^. Regardless of the exact type of the unit cell distortion, the anisotropy response due to strain engineering appears to be governed by the magnetoelastic constant as shown in Fig. 2f.

The frequency versus resonance field dependence for a perpendicularly applied magnetic field (Fig. 2b), yields the equivalent values of $M_{{{\text{eff}}}}$ as for the IP applied field within 8% margin of error. However, the fitting according to the Kittel equation for the OP oriented magnetic field:

4 $$f = \frac{{\mu _{0} \gamma }}{{2\pi }}\left( {H + H_{c} - M_{{{\text{eff}}}} } \right),$$

reveals an unexpected, U-shaped dependency in the fitting residuals provided by the high accuracy of the FMR technique (Fig. 2c). It should be highlighted that the measurements were carried out at the magnetic fields $~H > M_{{{\text{eff}}}}$, which are sufficient enough to saturate the magnetization following Stoner-Wohlfarth free energy model and we estimate that the precision of the magnetic field alignment with the film normal is better than $1^\circ$. The U-shaped dependency is not observed for the film deposited on GGG substrate, therefore, it might be ascribed as a resultant of a strain inhomogeneity.

**Figure 3:**
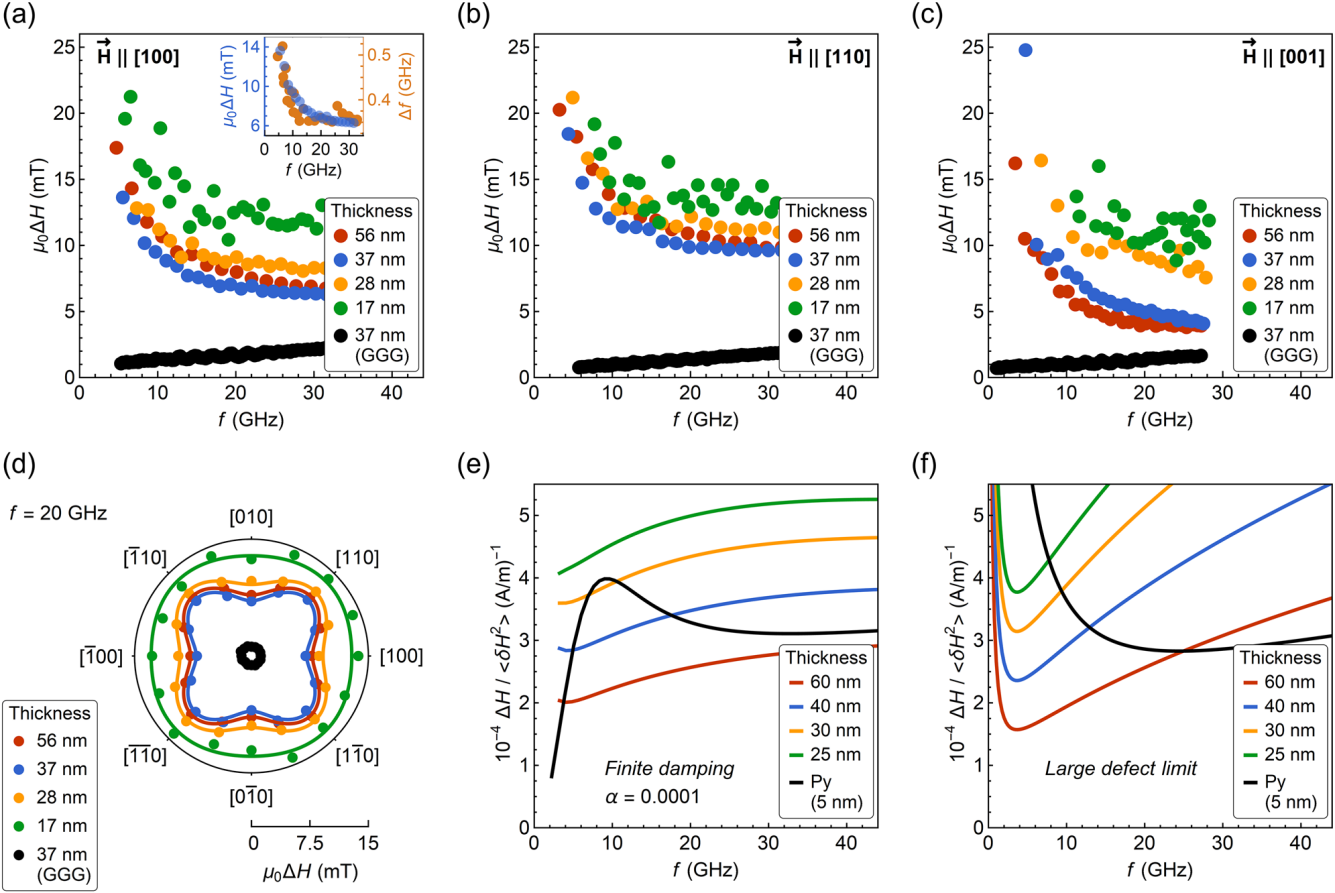
(**a**)-(**c**) Linewidth dependence on frequency for different orientations of the magnetic field with respect to crystallographic directions. The inset in (**a**) shows a comparison of linewidth changes for the field- and frequency-swept measurements of 37 nm thick film. (**d**) In-plane angular dependence of linewidth recorded at a constant frequency $f =$ 20 GHz. (**e**) and (**f**) Two-magnon contribution to the linewidth as a function of frequency calculated for finite damping (**e**) and large defect limit (**f**). The color lines correspond to films with a saturation magnetization of $M_{s} =$ 120 kA/m, while the black line denoted as “Py”, corresponds to a film with $M_{s} =$ 800 kA/m.

The FMR linewidths (FWHM) for the films deposited on YAG are significantly increased compared to the reference film deposited on GGG, and the data vaguely suggests the two-magnon scattering (TMS) contribution. As shown in Fig. 3a-c, the linewidth $\Delta H$ noticeably reduces for OP measurement configuration (${{\vec{H}}}||\left[ {001} \right]$) with respect to the IP measurements carried out for ${{\vec{H}}}||\left[ {100} \right]$ or ${{\vec{H}}}||\left[ {110} \right]$. Although this can be seen for the two thickest films, it is however not observed for the 28 and 17 nm thick layers for which one can expect the effect to be more expressed^40,41^. Another indication of TMS presence (usually considered as a characteristic) are the apparent variations in the IP angular analysis of the linewidth exhibiting fourfold symmetry (Fig. 3d). The TMS contribution is given by:

5 $$\Delta H_{{2mag}} \propto \Gamma _{4} ~\cos ~4\left( {\theta _{H} - \theta _{4} } \right)~\arcsin \frac{f}{{\sqrt {f^{2} + f_{0}^{2} } + f_{0} }},$$

where $f_{0} = \mu _{0} \gamma M_{{{\text{eff}}}}$ and $\theta _{4} =$ 45° is the angle of the maximum scattering rate^42–44^. The TMS strength coefficients ${{\Gamma }}_{4}$ for YIG/YAG films are of the order of ≈ 1.0 mT and relatively low in comparison to the magnitude of the linewidths ≈ 10 mT (Table 1). No systematic dependence of ${{\Gamma }}_{4}$ on thickness is observed, yet the magnitude of ${{\Gamma }}_{4}$ for the thinnest film is the lowest, contrary to the expectations provided by the TMS model.

For YIG/YAG films we observe an increase in linewidth broadening at low magnetic fields for all measured crystallographic directions, i.e., [100], [110], and [001] (Fig. 3a-c). The $\Delta H\left( f \right)$ dependencies are not linear and the slope of $\Delta H\left( f \right)$ is negative even at the highest frequencies, although the measurements were carried out at magnetic fields $H > M_{{{\text{eff}}}}$. Evaluation of the Gilbert damping parameter $\alpha$ is therefore not feasible according to:

6 $$\mu _{0} \Delta H = \alpha \frac{{4\pi }}{\gamma }f + \mu _{0} \Delta H_{0}.$$

Clearly, an extrinsic contribution dominates over the intrinsic Gilbert term in Eq. (6) up to frequencies of 30 GHz. To investigate if this behavior can be attributed to the so-called field dragging effect^45^, we have additionally performed frequency swept measurements. The result presented in the inset in Fig. 3a, shows that that frequency swept linewidth also increases at the low magnetic fields. Therefore, the broadening increase cannot be correlated with this kind of response, congruently with IP magnetization reversal curves from which we find magnetization saturation above 130 mT. For the reference film deposited on GGG, we determine $\alpha =$ (5.4 ± 0.6) × 10^‒4^ and the inhomogeneous linewidth broadening $\mu _{0} \Delta H_{0} =$ 0.72 ± 0.19 mT.

The abovementioned TMS contribution may suggest that the increase in linewidth broadening at low magnetic fields is also a result of TMS processes. The classical model of extrinsic FMR linewidth predicts such an increase for a 5 nm thick film with the saturation magnetization of 800 kA/m, exchange length of 5 nm, and the defect size of 100 nm^41^. In Fig. 3e,f, the linewidth broadening for such a case is displayed with a black line for finite damping $\alpha =$ 1 × 10^‒4^ and large defect limit, respectively. The calculations were based on the Eq. 28 and 42 in Ref.^41^. However, for much smaller saturation magnetization (120 kA/m) and exchange length of 17 nm for YIG, the model predicts qualitatively opposite changes in linewidth for film thicknesses ranging from 25 to 60 nm (Fig. 3e,f). For magnetic parameters of YIG, the linewidth increases for frequencies above 3 GHz. This suggests that another extrinsic effect is responsible for the linewidth broadening at low magnetic fields. The modeling for such contribution is proposed in the following section.

### Anisotropy dispersion model of FMR linewidth

We consider a thin film that is comprised of a set of non-interacting regions and assume that the inhomogeneous strain leads to the dispersion of uniaxial out-of-plane anisotropy, i.e., the magnitude of the anisotropy field vary in different regions and the anisotropy axis is tilted from the film normal. The cubic magnetocrystalline anisotropy is neglected to investigate the effect coming only from the uniaxial anisotropy. The analysis aims to examine resonant frequency distributions arising from the dispersion of uniaxial anisotropy for such sectioned thin film and to investigate if it can provide a qualitative agreement with the experiment.

**Figure 4:**
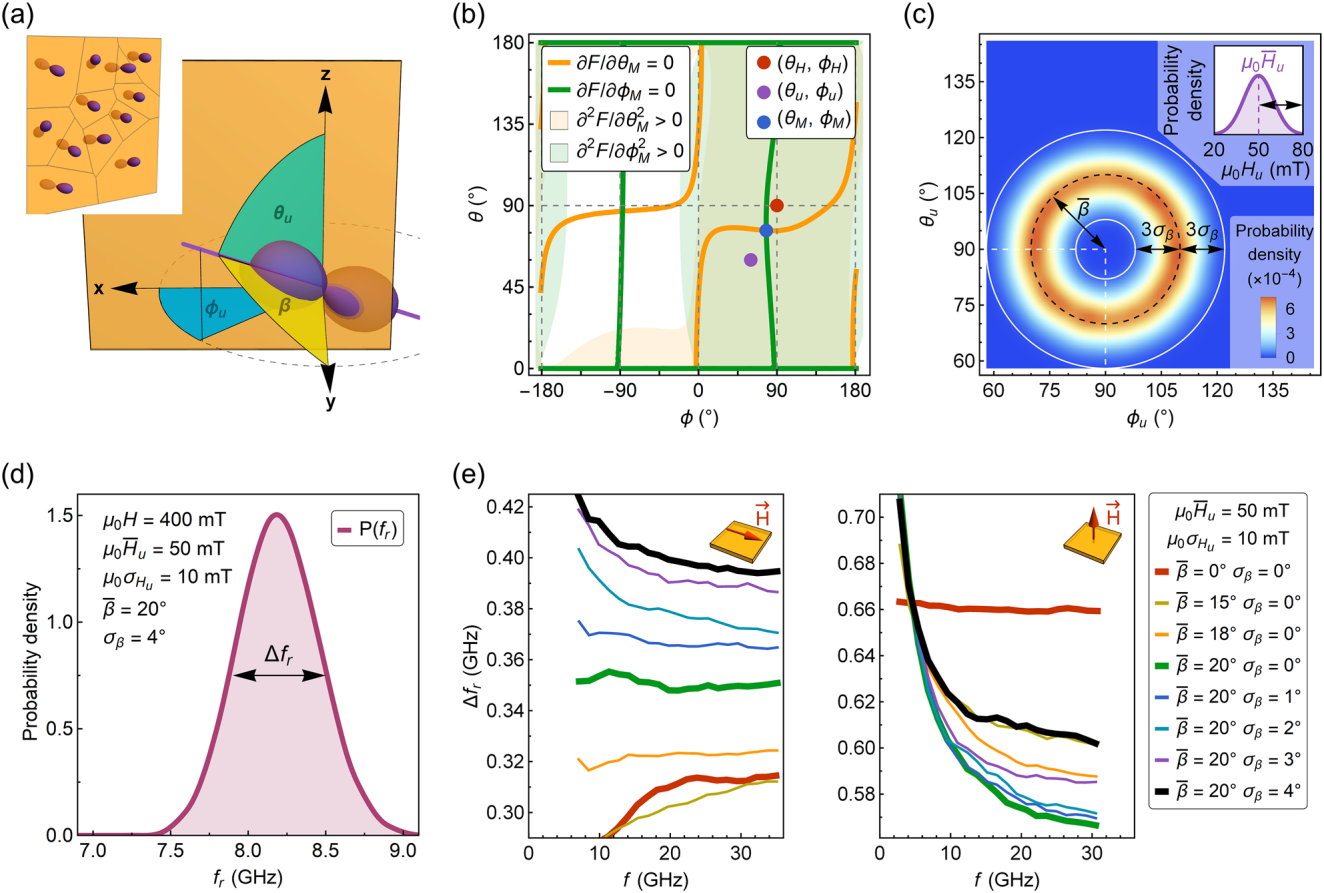
(**a**) Coordinate system used to describe the orientation of uniaxial anisotropy axis, the magnetization, and the applied field. Film lies in the x–z plane, normal to the film is along the y-direction. Inset schematically shows a film comprised of noninteracting regions with different orientations of uniaxial anisotropy axis. (**b**) Equilibrium orientation of magnetization determined from the condition of the free energy minimum with a tilted axis of uniaxial anisotropy. The example is given for the OP applied magnetic field $\mu _{0} H =$ 200 mT. (**c**) Gaussian distribution of the uniaxial anisotropy axis orientation. Inset shows the Gaussian distribution of the uniaxial anisotropy field and the arrows mark three standard deviations from the mean value (3 $\sigma _{{H_{u} }}$). (**d**) Exemplary distribution of the calculated resonance frequencies for the out-of-plane magnetic field $\mu _{0} H =$ 400 mT. (**e**) FWHM linewidth $\Delta f_{r}$ as a function of frequency for the in-plane applied magnetic field and the out-of-plane applied magnetic field (see the insets). All dependencies were calculated assuming $\bar{H}_{u} =$ 50 mT and $\sigma _{{H_{u} }} =$ 10 mT. The plot legend on the right side is valid for both IP and OP applied magnetic field. For $\bar{\beta } =$ 0° and $\sigma _{\beta } =$ 0° (red line), a constant broadening of the linewidth for OP magnetic field orientation and decreasing linewidth at low magnetic fields for IP orientation is observed. The tilting of the uniaxial anisotropy axis specified by $\bar{\beta } =$ 20° (green line) results in constant broadening and decreasing linewidth for IP and OP magnetic field, respectively. The decreasing linewidth as a function of frequency is found for both IP and OP magnetic field when $\sigma _{\beta } >$ 0°.

For each region of the film, the total free energy $F,$

7 $$F = F_{{{\text{Zee}}}} + F_{d} + F_{u}^{*} ,$$

includes the Zeeman energy $F_{{{\text{Zee}}}}$, the demagnetizing energy $F_{d}$ and the uniaxial anisotropy energy $F_{u}^{*}$, which axis orientation is defined by the angles $\theta _{u}$ and $\phi _{u}$ as shown in Fig. 4a. For certain values of $H_{u}$, $\theta _{u}$ and $\phi _{u}$, the magnetization orientation (expressed by the angles $\theta _{m}$ and $\phi _{m}$), is numerically calculated from the minimum of free energy: $\partial F/\partial \theta _{M} = 0$ and $\partial F/\partial \phi _{M} = 0$. An example illustrating a solution for these conditions is shown in Fig. 4b. Subsequently, a resonant frequency $f_{r}$ for a single film region is derived from the Smit-Beljers equation (see Eq. S6 in the Supplementary Information).

The resonant frequency distribution ${\text{P}}\left( {f_{r} } \right)$ with FWHM linewidth $\Delta f_{r}$, as shown in Fig. 4d, is obtained by taking into account the dispersion of uniaxial anisotropy:

1. $H_{u}$ varying according to Gaussian distribution with a mean value of $\bar{H}_{u} =$ 50 mT and standard deviation $\sigma _{{H_{u} }} =$ 10 mT (inset in Fig. 4c);
2. tilting of uniaxial anisotropy axis that is described with Gaussian distribution of angle $\beta$ with a mean value $\bar{\beta }$ and standard deviation $\sigma _{\beta }$ (Fig. 4c), since experimental results have not indicated any induced in-plane anisotropy, other than magnetocrystalline, cubic anisotropy.

The angle $\beta$ is related to $\theta _{u}$ and $\phi _{u}$ by $\cos \beta \equiv \sin \theta _{u} \sin \phi _{u}$. To probe the distributions of $H_{u}$ and $\beta$, we use 10^5^ sampling points and exclude the values that are larger or smaller than three standard deviations from the mean value. For the calculations, we have taken the magnetization saturation $M_{s} =$ 120 kA/m.

A resonant line ${\mathcal{L}}\left( f \right)~$ for a film comprised of non-interacting regions can be derived from:

8 $${\mathcal{L}}\left( f \right) = \int {\text{~P}}\left( {f_{r} } \right){\text{~L}}\left( {f - f_{r} ~} \right){\text{~}}df_{r} ,$$

where

9 $${\text{L}}\left( {f - f_{r} ~} \right) \propto \frac{{\left( {\frac{1}{2}~\Delta f} \right)^{2} }}{{\left( {f - f_{r} } \right)^{2} + \left( {\frac{1}{2}~\Delta f} \right)^{2} }}$$

is the Lorentz function, in which $\Delta f$ is the FWHM linewidth related to the Gilbert damping parameter $\alpha$:

10 $$\Delta f = \alpha \frac{{4\pi }}{{\mu _{0} \gamma }}f\frac{{\partial f}}{{\partial H}}.$$

In the limiting case of $\Delta f \to 0$, the FWHM of ${\mathcal{L}}\left( f \right)$ tends to $\Delta f_{r}$, and vice versa, when $\Delta f_{r} \to 0$, the FWHM of ${\mathcal{L}}\left( f \right)$ tends to $\Delta f$. As we have not experimentally observed Gilbert damping contribution in resonance linewidth dependence on frequency for YIG/YAG films, we conclude that the FWHM linewidth is described primarily by $\Delta f_{r}$.

**Figure 5:**
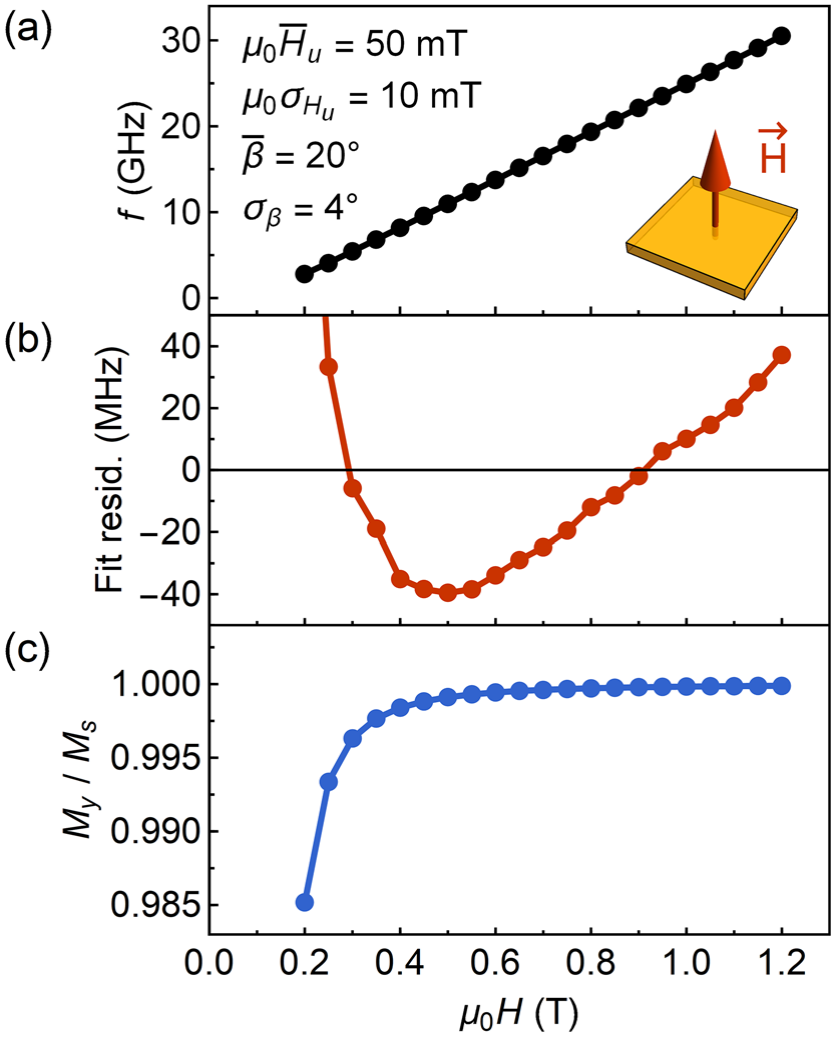
(**a**) Calculated Kittel relation for the out-of-plane magnetic field. Gaussian distributions of uniaxial anisotropy field $H_{u}$ and angle $\beta$ are specified inside the plot. (**b**) Fit residuals obtained from a fitting of dependency shown in (**a**) with Eq. (4). (**c**) Out-of-plane magnetization component as a function of the magnetic field.

The $\Delta f_{r}$ dependency on frequency obtained within this model agree well with our experimental results. For $\bar{\beta } =$ 20° and $\sigma _{\beta } >$ 0°, the increase in linewidth at low magnetic fields is found for both in-plane and out-of-plane applied magnetic fields (Fig. 4e). This strongly suggests that the axis of the strain-induced anisotropy for YIG/YAG films is tilted from the perpendicular direction and dispersed. Moreover, the linewidth broadening due to the dispersion of the anisotropy has a semiquantitative agreement with the experiment. The values of $\Delta f_{r}$ ≈ 0.3–0.7 GHz are of the same range of magnitude as the determined linewidth for YIG/YAG samples ≈ 0.34–0.52 GHz (see inset in Fig. 3a). Furthermore, the model predicts a similar type of discrepancy between calculated $f\left( H \right)$ dependence and the Kittel equation for OP resonance measurements (Fig. 5a,b). The fit residuals obtained from a fitting with Eq. (4), exhibit a comparable U-shaped dependency as found experimentally (Fig. 2c). At the same time, the OP magnetization component $M_{y}$ (mean over the film regions) displays only a minuscule decrease with decreasing magnetic field (Fig. 5c). This suggests that the tilting of magnetization can be hardly observed in the magnetization reversal curves at magnetic fields $H > M_{{{\text{eff}}}}$.

The main limitation of the proposed model comes from the assumption of non-interacting regions. Therefore, it does not provide accurate predictions at the magnetic fields close to the magnetization reversal, which in the FMR experiment, were not accessible due to large linewidth and consequently lack of FMR signal. Accordingly, the modeling was carried out at the field range from 0.2 to 1.2 T. Exchange and dipole interactions within and between the grains^46,47^, the distribution of uniaxial anisotropy at a certain correlation length would be crucial for quantitative estimation of remanent magnetization $M_{r}$ as well as for theoretical investigation of the magnetization reversal process. The proposed model can thus provide overestimated values of $M_{r}$ and coercive field. Notwithstanding the foregoing, in hysteresis loops of YIG films deposited on YAG, we clearly observe the decreased remanence and increased coercivity.

## Conclusions

In summary, the recrystallization of YIG on the lattice-mismatched substrate can result in different structural and magnetic properties when compared to the high-temperature deposition. As a consequence of the tensile strain, the positive values of the out-of-plane anisotropy field have been determined paving a path toward the attainment of the much-desired perpendicular anisotropy. We anticipate that, as shown here, the film growth conditions and strain control may play a significant role in the anisotropy tuning of other garnets as well. The mechanisms of the strain relaxation can lead to unexpected unit cell distortions, especially when inferred solely from a direct comparison of film and substrate lattice parameters. Moreover, the strain has a significant impact on FMR linewidth. For YIG/YAG films, the linewidth has been found noticeably increased, and the $\Delta H\left( f \right)$ dependency is characterized by an unusual negative slope across the entire frequency range from 5 to 30 GHz. The linewidth behavior was explained within the anisotropy dispersion model. Finally, good agreement of experimental findings with theoretical predictions suggests that the anisotropy axis is tilted from the film normal and dispersed. Therefore, we conclude that the strain homogeneity plays a crucial role in the attainment of narrow FMR linewidths reflecting low magnetization damping of the films.

## Methods

YIG films with thicknesses ranging from 9.6 nm to 56 nm were deposited at room temperature onto (001)-oriented Y_3_Al_5_O_12_ (YAG) substrates using the pulsed laser ablation technique (Nd:YAG laser, 355 nm). The pulse frequency of 2 Hz yielded a growth rate of ≈ 0.65 nm/min. The base pressure of the vacuum chamber was 8 × 10^‒6^ Pa (8 × 10^‒8^ mbar) and the partial pressure of oxygen was set to 2.4 × 10^‒2^ Pa (2.4 × 10^‒4^ mbar). After the deposition, the films were annealed *ex-situ* in air for 3 h at 800 °C. All samples were treated in a single annealing process for proper comparison. For reference, a 37 nm film was simultaneously deposited on a YAG substrate and a (001)-oriented Gd_3_Ga_5_O_12_ (GGG) substrate and reconstructed in the same process.

The structural properties of the films were investigated using high-resolution X-ray diffraction (XRD) with a four-crystal Ge (220) monochromator. The 2Theta/Omega scans allowed for a direct determination of the out-of-plane lattice parameters. The nominal values of film thicknesses were confirmed with X-ray reflectivity measurements. The surface morphology was examined with atomic force microscopy (AFM). The magnetic properties were investigated with a vibrating sample magnetometer (VSM) and broadband ferromagnetic resonance (VNA-FMR). All measurements were performed at room temperature and all uncertainties are one standard deviation unless otherwise noted.

## Supplementary Information

Supplementary Information.
