# Supplementary material for: Effect of strain-induced anisotropy on magnetization dynamics in Y3Fe5O12 films recrystallized on a lattice-mismatched substrate
Source: Sci Rep. 2021 Jul 7;11:14011. doi: 10.1038/s41598-021-93308-3 (PMC8263763; doi:10.1038/s41598-021-93308-3)
Supplement: Supplementary file 1 — Supplementary Information. [file 41598_2021_93308_MOESM1_ESM.pdf]

Supplementary Information

Effect of strain-induced anisotropy on magnetization dynamics in $Y_3Fe_5O_{12}$ films recrystallized on a lattice-mismatched substrate

Adam Krysztofik[1*], Sevgi Özoğlu[2,3], Robert D. McMichael[4], Emerson Coy[5]

[1] *Institute of Molecular Physics, Polish Academy of Sciences, ul. Smoluchowskiego 17, 60-179 Poznań, Poland*

[2] *Faculty of Physics, Adam Mickiewicz University, Uniwersytetu Poznanskiego 2, 61-614 Poznan, Poland*

[3] *Department of Physics, Graduate School of Natural and Applied Sciences, Hakkari University, Hakkari, Turkey*

[4] *National Institute of Standards and Technology, Gaithersburg, MD 20899, USA*

[5] *NanoBioMedical Centre, Adam Mickiewicz University, ul. Wszechnicy Piastowskiej 3, 61-614 Poznań, Poland*

* Corresponding author: adam.krysztofik@ifmpan.poznan.pl

- Derivation of Kittel equations
- X-ray diffraction pattern of 9.6 nm thick YIG film on YAG substrate
- Typical FMR spectra of YIG/YAG and YIG/GGG films

Derivation of Kittel equations

Free energy density for (001) oriented films with lattice distortion is defined as:

$$F = F_{\text{Zee}} + F_d + F_u + F_c, \tag{S1}$$

$$F_{\text{Zee}} = -\mu_0\, H\, M_s\, (\sin\theta_M \sin\theta_H \cos(\phi_H - \phi_M) + \cos\theta_M \cos\theta_H)\,, \tag{S2}$$

$$F_d = \frac{1}{2}\, \mu_0\, M_s^2 \sin^2\theta_M \sin^2\phi_M, \tag{S3}$$

$$F_u = -\frac{1}{2}\, \mu_0\, H_u\, M_s \sin^2\theta_M \sin^2\phi_M, \tag{S4}$$

$$F_c = \frac{1}{2}\, \mu_0\, H_c\, M_s \left(\alpha_x^2\alpha_y^2 + \alpha_y^2\alpha_z^2 + \alpha_z^2\alpha_x^2\right), \tag{S5}$$

where F_{Zee} is Zeeman energy term, F_d is the demagnetization energy term, F_u is the uniaxial out-of-plane anisotropy term, F_c is the magnetocrystalline cubic anisotropy term and α_x, α_y, α_z are the direction cosines. Expressions of F_d and F_u are defined for a film placed in the x-z plane. To derive Kittel equations, one can use the Smit-Beljers equation:

$$\left(\frac{\omega}{\gamma}\right)^2 = \frac{1}{M_s^2 \sin^2\theta_M}\left(F_{\theta\theta}F_{\phi\phi} - F_{\theta\phi}^2\right), \tag{S6}$$

however, to avoid an angular-dependent mixing it is more convenient to use the expression with Baselgia correction [1]:

$$\left(\frac{\omega}{\gamma}\right)^2 = \frac{1}{M_s^2}\left[F_{\theta\theta}\left(\frac{F_{\phi\phi}}{\sin^2\theta_M} + \frac{\cos\theta_M}{\sin\theta_M}F_\theta\right) - \left(\frac{F_{\theta\phi}}{\sin\theta_M} - \frac{\cos\theta_M}{\sin\theta_M}\frac{F_\phi}{\sin\theta_M}\right)^2\right], \tag{S7}$$

where

$$\begin{aligned} F_\theta &= \frac{\partial F}{\partial \theta_M} \qquad F_{\theta\theta} = \frac{\partial^2 F}{\partial \theta_M^2} \\ F_\phi &= \frac{\partial F}{\partial \phi_M} \qquad F_{\phi\phi} = \frac{\partial^2 F}{\partial \phi_M^2} \\ F_{\theta\phi} &= \frac{\partial^2 F}{\partial \theta_M \partial \phi_M} \end{aligned} \tag{S8}$$

and $\omega = 2\pi f$ is the angular frequency, γ is the gyromagnetic ratio.

For the in-plane applied magnetic field ($\phi_H = 0°$), Eq. S7 takes the form:

$$f = \frac{\mu_0 \gamma}{2\pi} \sqrt{(H + H_c \cos 4\theta_H)\left(H + \frac{1}{4} H_c (3 + \cos 4\theta_H) + M_{\text{eff}}\right)}, \qquad \text{(S9)}$$

while for the out-of-plane applied magnetic field ($\theta_H = 90°, \phi_H = 90°$) it yields:

$$f = \frac{\mu_0 \gamma}{2\pi} (H + H_c - M_{\text{eff}}), \qquad \text{(S10)}$$

where $M_{\text{eff}} = M_s - H_u$ is the effective magnetization.

References

1. Baselgia, L. *et al.* Derivation of the resonance frequency from the free energy of ferromagnets. *Phys. Rev. B* **38**, 2237–2242 (1988).

X-ray diffraction pattern of 9.6 nm thick YIG film on YAG substrate

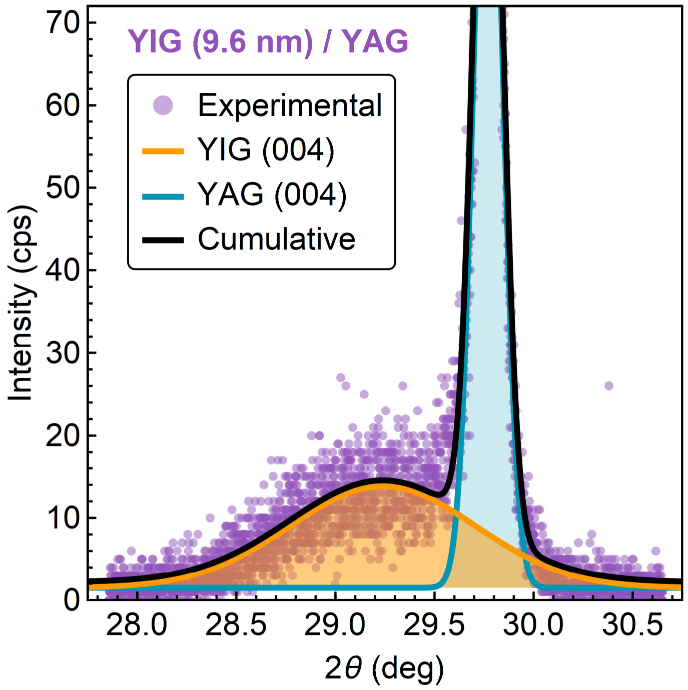

Fig. 1. X-ray diffraction pattern of 9.6 nm thick YIG film.

Typical FMR spectra of YIG/YAG and YIG/GGG films

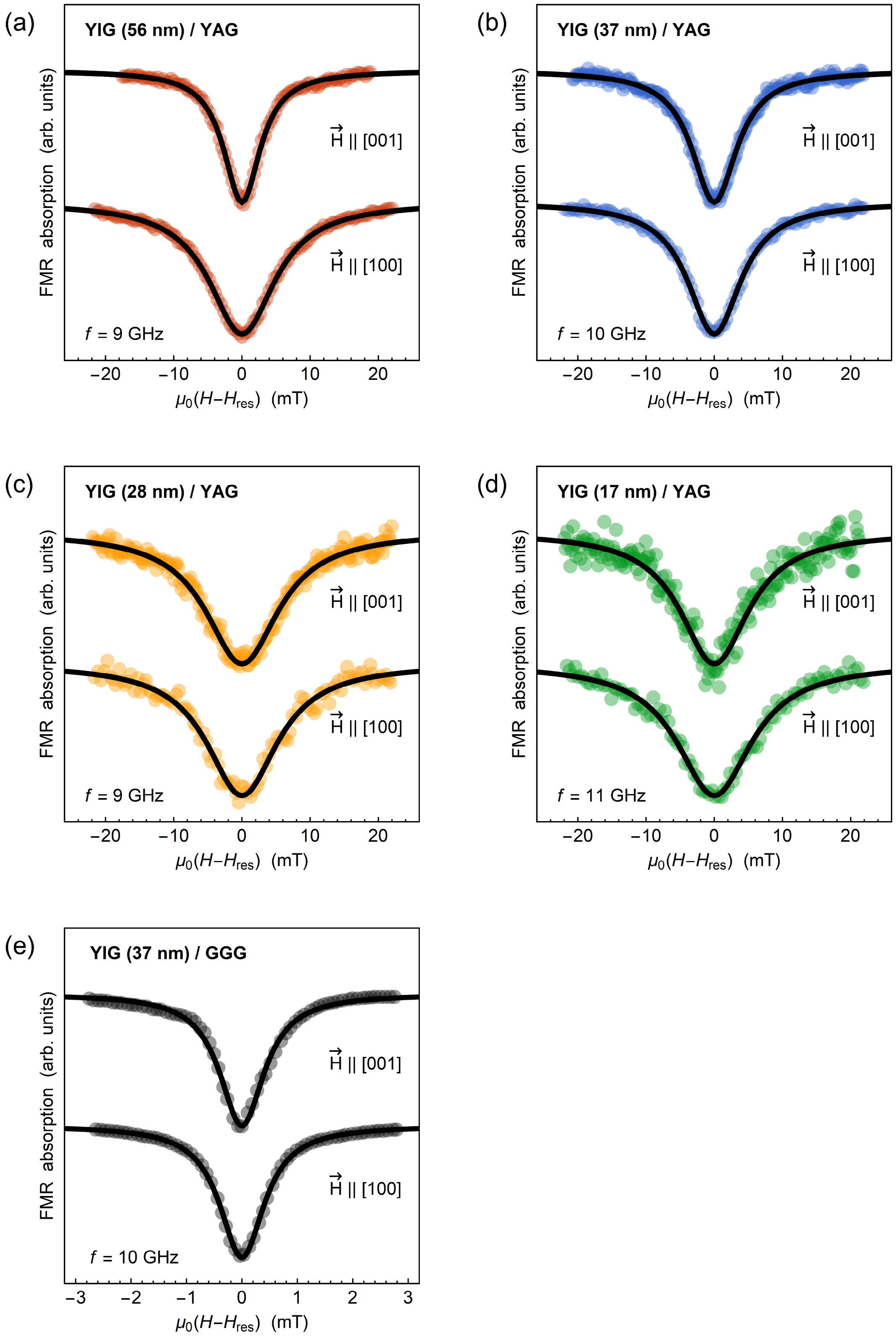

Fig. 2. (a)-(e) Typical FMR spectra taken at $f \approx 10$ GHz.
